# Return of results in the genomic medicine projects of the eMERGE network

**DOI:** 10.3389/fgene.2014.00050

**Published:** 2014-03-26

**Authors:** Iftikhar J. Kullo, Ra'ad Haddad, Cynthia A. Prows, Ingrid Holm, Saskia C. Sanderson, Nanibaa' A. Garrison, Richard R. Sharp, Maureen E. Smith, Helena Kuivaniemi, Erwin P. Bottinger, John J. Connolly, Brendan J. Keating, Catherine A. McCarty, Marc S. Williams, Gail P. Jarvik

**Affiliations:** ^1^Division of Cardiovascular Diseases, Mayo ClinicRochester, MN, USA; ^2^Cincinnati Children's Hospital Medical CenterCincinnati, OH, USA; ^3^Boston Children's HospitalBoston, MA, USA; ^4^Department of Genetics and Genomic Sciences, Charles R. Bronfman Institute for Personalized Medicine, Icahn School of Medicine at Mount SinaiNew York, NY, USA; ^5^Center for Biomedical Ethics and Society, Department of Pediatrics, Vanderbilt University School of MedicineNashville, TN, USA; ^6^Biomedical Ethics Program, Mayo ClinicRochester, MN, USA; ^7^Feinberg School of Medicine, Northwestern UniversityChicago, IL, USA; ^8^The Sigfried and Janet Weis Center for Research, Geisinger Health SystemDanville, PA, USA; ^9^Center for Applied Genomics, Abramson Research Center, The Children's Hospital of PhiladelphiaPhiladelphia, PA, USA; ^10^Research Division, Essentia Institute of Rural HealthDuluth, MN, USA; ^11^Genomic Medicine Institute, Geisinger Health SystemDanville, PA, USA; ^12^Departments of Medicine (Medical Genetics) and Genome Sciences, University of WashingtonSeattle, WA, USA

**Keywords:** genomics, electronic health records, incidental findings, implementation, genetic counseling, next generation sequencing, pharmacogenetics

## Abstract

The electronic Medical Records and Genomics (eMERGE) (Phase I) network was established in 2007 to further genomic discovery using biorepositories linked to the electronic health record (EHR). In Phase II, which began in 2011, genomic discovery efforts continue and in addition the network is investigating best practices for implementing genomic medicine, in particular, the return of genomic results in the EHR for use by physicians at point-of-care. To develop strategies for addressing the challenges of implementing genomic medicine in the clinical setting, the eMERGE network is conducting studies that return clinically-relevant genomic results to research participants and their health care providers. These genomic medicine pilot studies include returning individual genetic variants associated with disease susceptibility or drug response, as well as genetic risk scores for common “complex” disorders. Additionally, as part of a network-wide pharmacogenomics-related project, targeted resequencing of 84 pharmacogenes is being performed and select genotypes of pharmacogenetic relevance are being placed in the EHR to guide individualized drug therapy. Individual sites within the eMERGE network are exploring mechanisms to address incidental findings generated by resequencing of the 84 pharmacogenes. In this paper, we describe studies being conducted within the eMERGE network to develop best practices for integrating genomic findings into the EHR, and the challenges associated with such work.

## Introduction

The availability and reduced costs of high-density genotyping and genome sequencing technologies has accelerated genomic discovery (Green et al., 2011). Genome-wide association studies (GWAS) have revealed numerous common genetic variants that influence susceptibility to disease and adverse drug reactions, as well as inter-individual variation in quantitative traits and drug response (Manolio, 2013). Next-generation sequencing has also enabled discovery of variants associated with rare heritable diseases (Yang et al., 2013). Assessing the utility and identifying best practices for integration of this new genomic knowledge into clinical practice to improve patient care is now a major focus in the area of translational genomics (Kullo et al., 2013; Manolio et al., 2013).

The electronic Medical Records and Genomics (eMERGE) network (see Supplementary Figure) was established in 2007 with support from the National Human Genome Research Institute (NHGRI) to further genomic discovery using biorepositories linked to the electronic health record (EHR). The initial phase (Phase I) of the eMERGE network included five sites: Group Health Cooperative/University of Washington, Marshfield Clinic, Mayo Clinic, Northwestern University, and Vanderbilt University. The network was successful in leveraging the EHR to discover new genetic associations including variants that influence traits such as hematologic traits, lipid levels, Alzheimer disease, and electrocardiographic intervals among many others (Kullo et al., 2010; Naj et al., 2011; Ding et al., 2012, 2013; Rasmussen-Torvik et al., 2012; Crosslin et al., 2013). Phase II of eMERGE began in August 2011 with the addition of Geisinger Health System and Mount Sinai Medical Center to the network. In August 2012, three pediatric sites joined the network: Children's Hospital of Philadelphia, and a joint membership of Cincinnati Children's Hospital Medical Center and Boston Children's Hospital. As was the case for Phase I sites, each new site has its own biorepository linked to clinical phenotypes obtained from the EHR. While continuing efforts at genomic discovery, eMERGE Phase II is also investigating ways of incorporating genomics into the clinical setting (Gottesman et al., 2013), in particular, the return of genomic results in the EHR for use by healthcare providers at the point of care.

Although the focus in Phase I of eMERGE was primarily on genomic discovery efforts, eMERGE investigators also reviewed what research findings should be considered for return to participants (Fullerton et al., 2012). A return of results working group was set up to address the types of genomic findings that might be returned to patients or participants in the future. The genetic data were generated from high-density genotyping arrays and the return of results working group considered two main categories of genomic findings for return to participants. The first related to common variants on these arrays that may have clinical utility, e.g., Factor V Leiden, and hemochromatosis *HFE* variants. The second related to sex chromosome abnormalities such as Klinefelter and Turner syndromes incidentally discovered on signal intensity analysis of fluorescent data from the genotyping arrays. The investigators agreed that the potential to change immediate medical care was an important criterion for considering return of a result. Based on this criterion, homozygosity of single nucleotide polymorphism (SNP) rs6025 (R506Q, also known as the Factor V Leiden mutation) and rs1800562 (*HFE* C282Y, associated with hereditary hemochromatosis) were judged to have the highest clinical relevance (Fullerton et al., 2012). Sex chromosome abnormalities were also considered for return to participants, although context, such as the age of the participant, was felt to be an important factor. The working group summarized these recommendations in a position paper (Fullerton et al., 2012).

In Phase II of eMERGE, genomic discovery efforts continue, but the focus has expanded to include clinical implementation of genomic data. In addition to high-density genotype data in a large number of patients (see Supplementary Table) (Gottesman et al., 2013), targeted next-generation sequencing data of 84 ‘very important pharmacogenes’ (VIPs) will be available for nearly 10,000 patients across the network as part of the eMERGE PGx project. Genetic data being considered for return to participants include SNPs of medical or pharmacogenetic relevance, genetic risk scores for common diseases as well as pharmacogenomic variants, and clinically actionable incidental findings related to next-generation sequencing of 84 VIPs (e.g., *RYR1* and *CACNA1S* genes associated with malignant hyperthermia). Candidate variants for possible return to participants and incorporation into EHR, will be validated in a CLIA-certified laboratory. Clinical integration of genomic data is being explored with active examination of the ethical, legal, and social implications of such integration for both patients and clinicians.

To start addressing the opportunities and challenges in returning genomic results in the clinical setting, each site is conducting genomic medicine pilot projects to return clinically relevant genomic results to participating patients and their health care providers. Additionally, pharmacogenomic information is being placed preemptively in the EHR as part of the network-wide eMERGE PGx project. In this article, we review the current status of the genomic medicine pilot projects at each eMERGE site and the challenges associated with such work. The aim of each project and the genetic variants being considered for return are summarized in Table 1. The categories of genomic results being considered for return to patients in eMERGE II include individual or multiplexed SNPs that influence disease susceptibility or drug responses. The following sections provide an overview of the activities in eMERGE II related to return of results in these pilot genomic medicine implementation projects. A separate manuscript is planned to address return of results in the setting of genomic discovery.

**Table 1:** **Summary of the eMERGE genomic medicine pilot projects**.

**Site**	**Study aim**	**Variants**	**Results returned by**	**EHR integration**
Essentia Health	Evaluate genetic markers for increased risk of age-related macular degeneration (AMD) in the clinical setting	Five variants in *CFH*, one variant in *ARMS-2*, one variant in *C3*, and one variant in *ND2*, will be genotyped and results returned	Optometrist	Provider is notified electronically that genetic results and risk score are available in patients' EHR
Geisinger Health System	(1) Incorporate *IL28B* genotyping and genotype-guided therapy into the standard treatment protocol for chronic hepatitis C virus infection (2) Develop a clinical WGS sequencing program. (3) Genetic risk score to identify patients for AAA screening	(1) Two variants in *IL28B* associated with treatment response (2) Identify causal variants based on indication for testing as well as clinically actionable incidental findings (3) Multi-SNP panel	(1) Hepatologist (2) Clinical geneticist and genetic counselor (3) Patient's provider, preventive care team	(1) Electronic Order Set (2) Genomic test report in laboratory section; certain variants (e.g. pharmacogenetic) will have CDS tied to computerized order entry (3) CDS tied to preventive health care reminder system
Group Health Cooperative and University of Washington	Six genes with highly penetrant variants will be sequenced and confirmed pathogenic variants will be returned to participants	Pathogenic variants in *CACNA1C* and *RYR1* (malignant hyperthermia), *KCNH2* and *SCN5A* (long QT syndrome), *RYR2* (catecholaminergic polymorphic ventricular tachycardia), and *LDLR* (hyperlipidemia)	Genetic counselor or medical geneticist	Results placed in the EHR
Mayo Clinic	Disclosure of genomic risk of myocardial infarction using a genetic risk score integrated into the Framingham risk score	28 SNPs associated with coronary heart disease in prior GWAS are genotyped in a CLIA laboratory and returned	Genetic counselor using an EHR-based tool followed by visit with a preventive cardiologist/internist	An EHR-based tool is used to communicate genomic risk. Genotyping results are placed in the EHR
Icahn School of Medicine at Mount Sinai	Evaluate implementation of a screening and decision support system for risk of non-diabetic kidney disease in African Ancestry patients with hypertension and/or family history of renal failure	*APOL1* risk allele status	Genetic counselor or primary care provider	Placed in the EHR and linked to CDS
Northwestern University	Assess the impact and use of genomic results on clinical care by both physicians and patients, to evaluate the use and impact of physician support documents and best practice alerts in the EHR for genomic results, and to evaluate the non-clinical care impact of genomic results on patients	Variants in FV, FII, and *HFE* mutations (*C282Y* and *H63D*)	Physician with referral to study genetic counselor available	Genomic test results available in laboratory section of EHR; Physicians alerted when results received; CDS developed to alert if risk is present; Contextual links to patient and physician information resources
Vanderbilt University	Three major outcomes are being assessed including the efficacy of pharmacogenomics testing in reducing adverse drug events, physician uptake, and patients' knowledge and reaction	Fourteen actionable genetic variants that include: CYP2C19 *2-*8 (clopidogrel), CYP2C9 *2-*3, VKORC1 rs9923231 (warfarin), SLCO1B1 *5 (simvastatin), and TMPT *1-*3 (thiopurines)	Ordering physician	Genomic test results available in laboratory section of EHR, Patient Summary, and in Patient Portal
Children's Hospital of Philadelphia	The main focus is on prevention of potentially life-threatening drug adverse events	HLA-B*1502 (carbamazepine induced Stevens-Johnson syndrome) and *TPMT* (thiopurines) variants	Ordering physician	Results shared with providers and placed in the EMR
Cincinnati Children's Hospital and Boston Children's Hospital	Explore parents' responses to their children's research results	Cincinnati Children's Hospital will return 21 variants of *CYP2D6* test results while Boston Children's Hospital will return hypothetical *CYP2D6* results	Genetics clinical nurse specialist	Results shared with primary care providers but not placed in EHR

AAA, abdominal aortic aneurysm; CDS, clinical decision support; GWAS, genome-wide annotation studies.

## Individual SNPs

Individual SNPs associated with disease risk or of pharmacogenomic relevance are being considered for return of results studies within the eMERGE network, and results of this type are being returned in two eMERGE II pilot studies. A variant in the apolipoprotein L1 (*APOL1*) gene that is associated with non-diabetic chronic kidney disease (CKD) in patients of African American ancestry (Tzur et al., 2010; Parsa et al., 2013) is being returned in a pilot study at the Mount Sinai Medical Center. To understand processes and the impact of implementing a screening and decision support system for risk of non-diabetic CKD in African American patients with hypertension and/or family history of renal failure, ~40 participants will be genotyped for three risk variants in exon 6 of *APOL1*. A catalog of current evidence-based guidelines for the management of hypertension and chronic kidney disease will group the participants into three renal care advice message categories including (i) evaluation of CKD, (ii) identification of CKD progression, and (iii) prevention of CKD progression. In-depth, audio-recorded qualitative interviews are being conducted with patients before and after they receive their *APOL1* results. Analysis of the transcribed interviews will inform the design of the clinical decision support and patient education materials, and the design of quantitative questionnaires for use in a planned larger study of returning *APOL1* results to patients in clinical practice.

At Northwestern University, investigators are following up on the recommendations from the Return of Results workgroup in eMERGE I to include return of potentially actionable findings by reconsenting 150 biobank participants who were genotyped during Phase I of eMERGE. Participants will undergo CLIA-certified genotyping of the Factor V Leiden mutation and the hereditary hemochromatosis *HFE* mutations (C282Y and H63D). Results will be deposited in the EHR and available to physicians. Study participants will be informed of their results via a letter that will be sent by post or accessible via an online patient web-portal called MyChart). Participants will also complete a baseline survey and follow-up surveys at 1 and 6 months after receiving results. Physicians and participants will also have the opportunity to discuss the results during an appointment or be referred to a study genetic counselor. Semi-structured interviews will be conducted with physicians after results are returned to patients and clinical decision support has been triggered in the EHR. The impact and use of genomic results on both physicians and patients will be assessed, as will the use and impact of physician support documents and best practice alerts in the EHR for genomic results.

## Pharmacogenetic variants

At the Vanderbilt University site, 14 pharmacogenetic variants including: *CYP2C19* ^*^2–^*^8 (clopidogrel), *CYP2C9* ^*^2–^*^3, *VKORC1* rs9923231 (warfarin), *SLCO1B1* ^*^5 (simvastatin), and *TMPT* ^*^1–^*^3 (thiopurines) will be genotyped in their study population. Vanderbilt University has conducted a number of research studies to assess the efficacy of the Pharmacogenomic Resource for Enhanced Decision in Care and Treatment (PREDICT) program in reducing adverse drug events, physician uptake, and patients' knowledge and attitudes toward such testing. Physician surveys and structured interviews have been performed. Interviews have been conducted in the following groups of patients who have been through the interventional cardiology clinic: (i) no medication change following PREDICT, (ii) a change in clopidogrel medication dosing, (iii) change in statin medication dosing, and (iv) not enrolled in PREDICT. Results of these surveys and interviews are awaited.

At the University of Washington-Group Health site, pathogenic variants in six highly penetrant pharmacogenes will be genotyped in ~450 participants. These variants include risk alleles in *CACNA1C* (malignant hyperthermia), *RYR1* (malignant hyperthermia), *KCNH2* (long QT syndrome), *SCN5A* (long QT syndrome), *RYR2* (catecholaminergic polymorphic ventricular tachycardia), and *LDLR* (hyperlipidemia). Results are expected to be returned through the Department of Clinical Genetics with appropriate counseling and subsequent documentation in the EHR. Patients will be surveyed regarding their experience. Healthcare providers will be surveyed to assess their use of genetic information in EHR, ease of use, completeness of information in the EHR, and whether any other resources are needed. Additionally, feasibility of implementing genomic clinical decision support will be assessed by interviews with patients and healthcare providers to guide development and testing of prototype interfaces for the EHR.

In a clinical implementation project at Geisinger Health System, all newly diagnosed patients with chronic HCV are genotyped in a CLIA laboratory for two variants in the interleukin 28B gene *(IL28B)* that influence treatment response and can influence medication choice. A genotype-guided treatment decision tree was created in consultation with expert clinicians and an electronic order set was implemented in the EHR to insure that all eligible patients underwent testing. Genotype results for any participant who is prescribed interferon alpha and ribavirin for chronic HCV infection are placed in the EHR and are available to hepatologists initiating treatment. The economic impact of use of these variants is being studied.

Investigators at Children's Hospital of Philadelphia are working on developing tools to engage local practitioners in targeted intervention projects trialing in-house web-based software that integrates with the EHR (Fiks et al., 2013). This new tool can be used to query the institutional biobank as well. So far, 515 patients have been genotyped for HLA-B^*^1502 which is associated with risk of developing Stevens-Johnson syndrome following use of carbamazepine (Chen et al., 2011), in addition to 318 patients genotyped for *TPMT* in patients that may be treated with thiopurines.

Investigators at Cincinnati Children's Hospital Medical Center are studying the return of *CYP2D6* variants in pediatric patients in the content of codeine response, and returning these results to the parents. Variants of *CYP2D6* are genotyped using Taqman and long polymerase chain reaction for full gene deletion and duplication. At the time results are returned to parents, they complete a telephone survey about their reactions and plans to share the actual results, and their anticipated preferences regarding receiving hypothetical incidental findings. Follow-up telephone calls are being conducted at 3 and 12 months post-result disclosure to learn how results were used. A subset of parents are also participating in qualitative interviews to further explore their reactions and perceptions to receiving their children's *CYP2D6* results. Although results will not be placed in the EHR, the researchers are asking parents for permission to share the results with their child's primary care providers and will evaluate primary care providers' reactions to receiving the results. At the Boston Children's Hospital site, the same study, including the same survey and qualitative interviews, is being carried out but using hypothetical, not actual *CYP2D6* results, and without the follow-up telephone calls. Providers will be surveyed about the perceived utility of pharmacogenomics research results in their practice. Between the two sites in Cincinnati and Boston, parents of 400 children will be enrolled. To date, Cincinnati Children's has returned 48 children's *CYP2D6* results to parents and has recontacted 21 parents, all of whom have given permission to share the results with their children's primary care providers.

## Genetic risk scores

Genetic findings of clinical relevance from high-density genotyping arrays include numerous common alleles that influence disease susceptibility. Because most individual variants have modest effect sizes, investigators are exploring the utility of combining the risk variants into genetic risk scores. At the Marshfield/Essentia Health site, a genetic risk score for age-related macular degeneration (AMD) is calculated based on five variants in *CFH*, one variant in *ARMS-2*, one variant in *C3* and one variant in *ND2*. Individuals (*n* = 100) attending optometry clinics are genotyped for these variants (Haines et al., 2005) and a genetic risk score is calculated and incorporated into the EHR by optometrists. Overall risk will be calculated from the genotypes and the patient's smoking status. The results will be shared with the study participants and a telephone survey will be conducted. The participating clinicians will be interviewed after reviewing these results. Recruitment for this study began in June 2013.

At Mayo Clinic, investigators are conducting the Myocardial Infarction Genes (MI-GENES) clinical trial, a pilot study of communicating genetic risk for coronary heart disease (CHD), the leading cause of death in the US. Patients at intermediate risk for CHD based on conventional risk factors will undergo genotyping of ~28 SNPs that are associated with CHD independent of lipid or blood pressure levels, in a CLIA laboratory (Deloukas et al., 2013). Study participants will be randomized to receive a Framingham risk score or a modified Framingham risk score that incorporates a genetic risk score based on the genotyping results. The genetic risk scores will be placed in the EHR and an EHR-based pictogram will be used to assist in discussing genomic risk of CHD. Patients in the study will be followed for at least 6 months to assess the extent to which there are differences in diet, physical activity, and other lifestyle modifications associated with the communication of CHD-related genomic risk information. Participants will also be evaluated for psychosocial and behavioral changes. Recruitment for MI-GENES started in October 2013.

At Geisinger Health System, investigators are combining clinical risk factors and genomic information to develop a risk score for abdominal aortic aneurysm (AAA), a leading cause of death in older men (Kent et al., 2010; Kuivaniemi et al., 2012). The goal is to develop strategies for population screening that combine genetic susceptibility variants with clinical risk factor data mined from EHR. Patients in the Geisinger MyCode biobank (Gerhard et al., 2013; Gottesman et al., 2013) will be genotyped for variants known to be associated with AAA (Kuivaniemi et al., 2013) and this information used to create a risk score that will then be evaluated in the Geisinger patient population after performing abdominal ultrasonography examination.

## Targeted next-generation sequencing

The eMERGE pharmacogenomics (PGx) project is using targeted sequencing of 84 pharmacogenes to initiate a multi-site test of the concept that genomic sequence information can be coupled to EHRs for use in the clinical setting (Gottesman et al., 2013). The PGRNseq uses a reagent to “capture” exonic sequences of 84 genes important in pharmacokinetic or pharmacodynamics processes for sequencing on next-generation platforms. Genotypes of established pharmacogenomics utility that influence use of simvastatin, clopidogrel, and warfarin will be confirmed in a CLIA environment and be placed pre-emptively in the EHRs of patients who are “at risk” of receiving these drugs.

Sequence information will also be generated for six genes for which the American College of Medical Genetics and Genomics (ACMG) guidelines suggest returning known or expected pathogenic variants given the association with highly penetrant actionable disorders (Green et al., 2013) (Table 2). These include genes associated with long QT syndrome genes *(KCNH2* and *SCN5A)*, malignant hyperthermia (*RYR1* and *CACNA1S*), hypercholesterolemia (*LDLR*), and catecholaminergic polymorphic ventricular tachycardia (*RYR2*).

**Table 2:** **Pharmacogenes being sequenced in the eMERGE PGx project and on the ACMG incidental finding list (Green et al., 2013)**.

**Gene**	**Phenotype**	Inheritance ^*^	Variants to report ^**^
*RYR2*	Catecholaminergic polymorphic ventricular tachycardia	AD	KP
*KCNQ1 KCNH2 SCN5A*	Romano-Ward long QT syndrome types 1, 2, and 3, Brugada syndrome	AD	KP and EP
*LDLR*	Familial hypercholesterolemia	SD	KP and EP
*RYR1 CACNA1S*	Malignant hyperthermia susceptibility	AD	KP

* SD, semi-dominant inheritance; AD, autosomal dominant.

** EP, expected pathogenic, sequence variation is previously unreported and is of the type that is expected to cause the disorder. KP, known pathogenic variants.

Adapted from the ACMG Policy Statement (Green et al., 2013).

ACMG guidelines emphasize return of mutations in these genes that are known to be pathogenic but also suggest returning novel mutations that are “expected” to be pathogenic. The recommendations have stimulated considerable debate (Green et al., 2013), and in particular, whether patient preferences can or should be incorporated into the return of results pipeline has been highlighted (Allyse and Michie, 2013). While the ACMG recommendations do not apply to research participants, members of the eMERGE network's Consent, Education, Regulation and Consultation (CERC) workgroup have weighed in with concerns about the scope of the ACMG position (Burke and Grefenstette, 2013; Ross et al., 2013). The mechanisms for whether and how to return incidental findings are being evaluated at each site. Several sites, in consultation with respective IRBs, are considering confirming pathogenic incidentally found variants for example in *RYR1* and *CACNA1S,* with an orthogonal genotyping method. This would be followed by inviting the study patient to a genetic counseling session during which the option of knowing the research results with confirmation in a CLIA lab will be discussed. Table 3 summarizes the approach of the various sites toward returning incidental findings generated as part of the eMERGE PGx project.

**Table 3:** **Return of results related to the eMERGE pharmacogenomics (PGx) projects at each of the eMERGE network sites**.

**Site**	**Genetic variants to be returned and placed in the EHR**	**Return of incidental findings of known clinical significance**
Geisinger Health System	Pharmacogenetic variants relevant to clopidogrel, warfarin, and simvastatin	IFs will be returned only if they have clear clinical significance.
Group Health Cooperative and University of Washington	PGx variants for carbamazepine sensitivity are approved for inclusion in the EHR	IFs will be returned by a clinical geneticist and this information would be placed in the EHR at the time of the encounter.
Marshfield Clinic	Pharmacogenetic variants relevant to clopidogrel, warfarin, and simvastatin	IFs will be returned only if they are clinically relevant based on input from a physician, clinical geneticist and/or medical specialist in that area of expertise.
Mayo Clinic	Pharmacogenetic variants relevant to clopidogrel, warfarin, and simvastatin	IFs will be reviewed by a multidisciplinary group prior to return.
Icahn School of Medicine at Mount Sinai	4 NYS/CLIA-approved genetic variants relevant to clopidogrel, warfarin, and simvastatin	IFs will not be returned.
Northwestern University	Pharmacogenetic variants relevant to clopidogrel, warfarin, and simvastatin	IFs will not be returned.
Vanderbilt University	11 PGx variants relevant to clopidogrel, warfarin, and simvastatin were already reported	IFs will not be returned.
Children's Hospital of Philadelphia	Variants in several pharmacogenes will be returned: *CYP2D6* and *UGT2B7* (codeine), *CRHR1* (fluticasone propionate), *UGT1A4* (lamotrigine), *KCNH2* (loratadine), *SLCO2B1* (montelukast, *CYP2D6*, *ABCB1*, *OPRM1*, *COMT*, and *UGT2B7* (morphine), *CYP2C19* and *AHR* (omeprazole), *ABCB1* (ranitidine), *ADRB2* (salbutamol), *BDNF* (sertraline), and *IL28B* and *HLA-DR/DQ* (interferon response variants)	IFs will be returned only if they are clinically relevant based on input from a physician, clinical geneticist and/or medical specialist in that area of expertise.
Cincinnati Children's Hospital (CCHMC) and Boston Children's Hospital (BCH)	CCHMC: Genetic variants in *CYP2D6* pre-emptively placed in EMR for children at risk for having surgery. PGx variants placed in EMR at point of care include those relevant for warfarin, thiopurines, tricyclic antidepressants and some SSRIs. BCH: Genetic variants relevant to warfarin	CCHMC: IFs need to be reviewed and approved by IRB before return and placement in EHR. BCH: IFs will be reviewed by Informed Cohort Oversight Board (ICOB) and appropriate action will be determined.

IFs, incidental findings.

## Whole genome/exome sequencing

Whole genome and exome sequencing are being increasingly utilized in the clinical setting (Yang et al., 2013). Although less than 2% of adults appear to have relevant actionable incidental findings from whole exome sequencing (Dorschner et al., 2013) whether to return such findings is an important topic of debate (Green et al., 2013), particularly in the context of preserving patient autonomy and confidentiality (Klitzman et al., 2013). Institutional committees with diverse expertise will need to adapt guidelines proposed by the ACMG to determine whether and how these can be applied locally, which results will be reported through local EHRs and what format these reports will take. The Clinical Sequencing Exploratory Research (CSER) consortium is also actively investigating return of results in studies utilizing whole genome or whole exome sequencing (https://cser-consortium.org). Several of the eMERGE sites have conducted pilot studies of whole genome/exome sequencing in the clinical setting. For example, at Geisinger Health System, investigators are developing a laboratory report that summarizes results of whole genome sequencing in individuals with intellectual disability and normal chromosomal microarray that have been recruited along with their parents to undergo whole genome sequencing to identify an underlying genetic etiology. Causal variants and incidental findings (from the ACMG list) will be validated using Sanger sequencing. All patients will be informed about the results and undergo counseling. Qualitative data from interviews will be collated and used to improve this process.

## Integration of genomic findings into the EHR

A recent theme issue of *Genetics in Medicine* addressed the issues related to integrating genomic findings into the EHR with linkage to clinical decision support at point of care (Kannry and Williams, 2013). Lack of standardized nomenclature for genetic variants is a major hurdle to creating automated decision support. Currently, several groups, including the Health Level 7 Genomics Work Group, are attempting to address this challenge. Additional challenges relate to a number of ethical, legal, and social implications that have been reviewed elsewhere (Hartzler et al., 2013; Hazin et al., 2013). These include uniform provision of genomic CDS to prevent worsening of disparities in healthcare, education of patients and providers, determining which genomic information to include in the EHR, managing incidental findings, privacy and documentation, storage and reinterpretation of genomic data and the results of stakeholder engagement in making these determinations. Education of patients and care providers will be necessary to facilitate the process of return of genomic results. The CERC Work Group in eMERGE has begun to address some of the education issues through jointly developing education materials and a website for patient information (www.myresults.org) and contributing to evaluation of online pharmacogenomics information for physicians. The CERC Working Group also has collaborative interactions with the CSER and Return of Results consortia.

## Summary

Genomic technology has advanced greatly through the last decade. As we attempt to implement genomic medicine, many challenges arise, including the need to develop suitable approaches to return results and to deal with incidental findings. We have summarized activities within the eMERGE network that are related to returning genomic results in the EHR setting and also with the return of incidental findings. Initial experiences within the eMERGE network highlight the need for additional studies that address the issues related to disclosure of results from genomic implementation studies. Ongoing work in the eMERGE network will provide important insights into best practices for returning genomic results and dealing with incidental findings using the EHR.

## Conflict of interest statement

The Review Editor Yiran Guo declares that, despite being affiliated to the same institution as authors John J. Connolly and Brendan J. Keating, the review process was handled objectively and no conflict of interest exists.
