# Supplementary material for: Return of results in the genomic medicine projects of the eMERGE network
Source: Front Genet. 2014 Mar 26;5:50. doi: 10.3389/fgene.2014.00050 (PMC3972474; doi:10.3389/fgene.2014.00050)

| **Supplementary Table.** Genotyped samples in the eMERGE network. | | |
| --- | --- | --- |
| **Site Sample Datasets** | **Genotyping Platform** | **n** |
| Boston Children’s Hospital | Affymetrix Axiom | 1024 |
| Cincinnati Children’s Hospital Medical Center | 610/660W/AffyA6/Omni1/Omni5 | 4322 |
| Children’s Hospital of Philadelphia | 550/610/Beadchi/AffyA6/AffyAxiom/OmniExpress | 6850 |
| Geisinger Health System | Illumina HumanOmni Express | 3111 |
| Group Health Cooperative/University of Washington | Illumina Human 660W Quad-v1 Illumina HumanOmni Express | 3520 |
| Icahn School of Medicine at Mount Sinai | Affymetrix 6.0 and Illumina HumanOmni Express | 6290 |
| Marshfield/Essentia Health | Illumina Human 660W Quad-v1 | 4809 |
| Mayo Clinic | Illumina Human 610 and 660W Quad-v1 | 6876 |
| Northwestern University | Illumina Human 660W Quad-v1 Illumina HumanOmni Express 12v1_C | 4858 |
| Vanderbilt University | Illumina Human 660W Quad-v1, Illumina HumanOmni Express 12v1_C, Omni 1, and Omni 5 | 13632 |
| **Total** |  | **55292** |

**Supplementary Figure.** Map of eMERGE network sites.


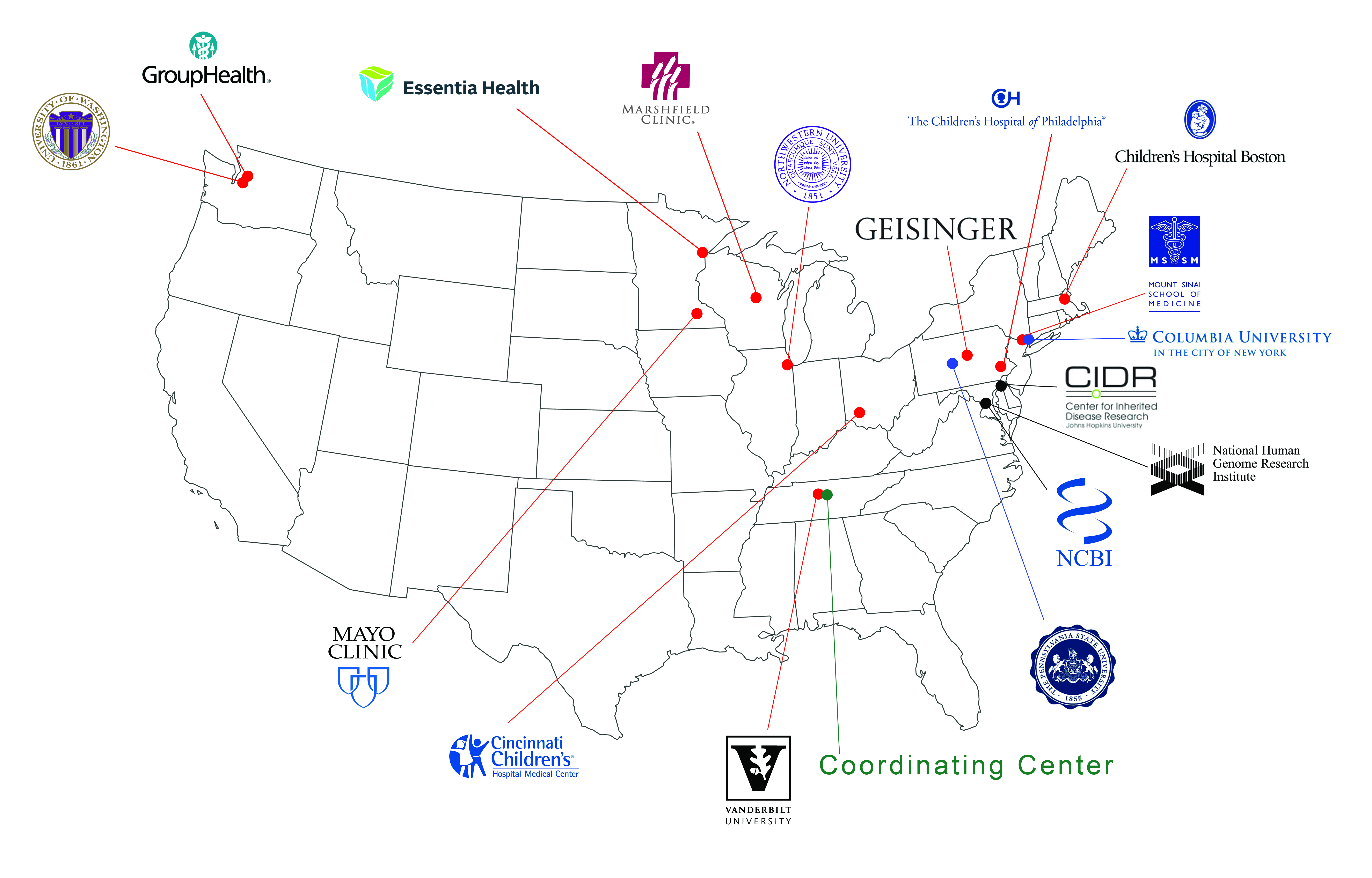

Supplement: Supplementary file 1 [file DataSheet1.DOCX]
